# Pharmacological Approaches That Slow Lymphatic Flow As a Snakebite First Aid

**DOI:** 10.1371/journal.pntd.0002722

**Published:** 2014-02-27

**Authors:** Dirk F. van Helden, Paul A. Thomas, Peter J. Dosen, Mohammad S. Imtiaz, Derek R. Laver, Geoffrey K. Isbister

**Affiliations:** 1 School of Biomedical Sciences & Pharmacy, University of Newcastle, Callaghan, New South Wales, Australia; 2 Hunter Medical Research Institute, New Lambton Heights, New South Wales, Australia; 3 School of Medicine, University of Queensland, Brisbane, Queensland, Australia; 4 Department of Nuclear Medicine, Royal Brisbane and Women's Hospital, Herston, Queensland, Australia; 5 School of Medicine and Public Health, University of Newcastle, Callaghan, New South Wales, Australia; 6 Department of Clinical Toxicology and Pharmacology, Calvary Mater Newcastle, Waratah, New South Wales, Australia; Liverpool School of Tropical Medicine, United Kingdom

## Abstract

**Background:**

This study examines the use of topical pharmacological agents as a snakebite first aid where slowing venom reaching the circulation prevents systemic toxicity. It is based on the fact that toxin molecules in most snake venoms are large molecules and generally first enter and traverse the lymphatic system before accessing the circulation. It follows on from a previous study where it was shown that topical application of a nitric oxide donor slowed lymph flow to a similar extent in humans and rats as well as increased the time to respiratory arrest for subcutaneous injection of an elapid venom (*Pseudonaja textilis*, Ptx; Eastern brown snake) into the hind feet of anaesthetized rats.

**Methodology/Principal Findings:**

The effects of topical application of the L-type Ca^2+^ channel antagonist nifedipine and the local anesthetic lignocaine in inhibiting lymph flow and protecting against envenomation was examined in an anaesthetized rat model. The agents significantly increased dye-measured lymph transit times by 500% and 390% compared to controls and increased the time to respiratory arrest to foot injection of a lethal dose of Ptx venom by 60% and 40% respectively. The study also examined the effect of Ptx venom dose over the lethal range of 0.4 to 1.5 mg/kg finding a negative linear relationship between increase in venom dose and time to respiratory arrest.

**Conclusions/Significance:**

The findings suggest that a range of agents that inhibit lymphatic flow could potentially be used as an adjunct treatment to pressure bandaging with immobilization (PBI) in snakebite first aid. This is important given that PBI (a snakebite first aid recommended by the Australian National Health and Medical research Council) is often incorrectly applied. The use of a local anesthetic would have the added advantage of reducing pain.

## Author Summary

Snakebite remains a major problem worldwide causing death or serious illness in many tens of thousands of victims annually. An approach to reduce the burden of envenoming is to provide optimum first aid procedures. We have previously shown that topical application of a nitric oxide (NO) donor slowed lymph flow to similar extent in humans and rats as well as increased the time to respiratory arrest by ∼50% for subcutaneous injection of eastern brown snake venom into the hind feet of anaesthetized rats. The present study examines the use of several other topical pharmacological agents that aim to slow venom toxins reaching the circulation through the lymphatic system. The study found that the agents examined were similarly effective to that previously found for the NO donor. The fact that one of these is a commonly used topical local anesthetic may be an ideal adjunct first aid, as it provides first aid while reducing pain.

## Introduction

Snake envenoming worldwide remains a major health problem with 20,000–94,000 deaths [1] and a morbidity of several 100,000 people per year [2]. Snake envenoming has been identified as a neglected tropical disease and there is a desperate need for improved treatment. First aid procedures are a major part of treating snake envenomings because envenomings nearly always occur away from hospitals leading to delays before antivenom can be administered.

In Australia, the only formally accepted snakebite first aid is pressure bandaging with immobilization (PBI) [3], [4]. Local pressure pad with compression, another mechanical method, is also seen as a potentially useful first aid [5], [6], [7]. PBI, which has long been endorsed by the National Health and Medical research Council of Australia against bites from Australian snakes, aims to limit venom entry into the circulation by preventing lymphatic transport without inhibiting arterial or venous blood flow. Both mechanical methods are based on the principle that snake toxin molecules are generally large and can't directly enter the circulation, but are readily taken up by the lymphatics [8]. To date these first aid procedures have been primarily applied for elapid snake bites of Australia and New Guinea as these venoms have limited local cytotoxicity with the main concern being death by actions once venoms enter the circulation [9].

It has been shown that PBI is often incorrectly applied particularly by untrained personnel, with reported success rates of 15% in untrained and 50% in trained personnel [10]. Thus, while PBI is highly effective at limiting toxin entry into the circulation in animal studies and mock venom studies [11], [12], its application in the clinical setting may be more tenuous. Therefore, co-application of adjunct methods that impede venom transport through the lymphatics may be beneficial.

Recently, we reported that topical application of a nitric oxide (NO) donor that is known to inhibit lymphatic pumping [13], as a potential first aid against snakebite. However, there are various other agents that are also known to inhibit spontaneous contractions of lymphatic smooth muscle and hence the intrinsic propulsion of lymph. It is therefore important to investigate whether such agents act in the same way as NO donors with the hope that they may be even more effective or provide a cheaper or more advantageous first aid. The present study compares the efficacy of the NO donor to several other topical agents that have known inhibitory actions on lymphatic function by directly or indirectly targeting the intrinsic lymphatic pump.

## Materials and Methods

### Ethics statement

All experiments were approved by the University of Newcastle Animal Care and Ethics Committee for ethics approval A-2009-153 according to the Australian Code of Practice for the care and use of animals for scientific purposes as released by the National Health and Medical Research Council of Australia in 2004.

### Experimental procedures

Studies were performed on urethane (1.5–1.75 g/kg i.p.) -anaesthetized male and female Wistar rats (weight range 200–550 g) at 37°C with animals euthanized without recovery at the end of the experiments. In some experiments groin lymphatic vessels were surgically exposed to facilitate measurement of foot to groin lymph transit times [13]. Envenoming was simulated by injection of snake venom (*Pseudonaja textilis*, Ptx; Eastern brown snake) into the feet of deeply anaesthetized rats. Our use of this venom in rats was not intended to mimic *P. textilis* venom action on humans, but was chosen because of its clear end point in the rat model of our studies. In the present studies we used three separate batches of freeze-dried Ptx venom (each obtained from pooled venom collected from 10–20 snakes). While two donated by the Australian Reptile Park (Somersby, NSW, Australia) showed very similar potency, the third batch from Venom Supplies (Adelaide) was less potent. Therefore the third batch was applied at proportionally higher concentrations to equate to the toxicity of the other two batches, as tested by trialing different doses until a dose was obtained that produced a similar time to respiratory arrest as for the first two batches for a dose of 1 mg/kg.

Experiments involved examining the effects of topical application of test pharmacological agents (nifedipine – Sigma N7634, lignocaine - Sigma L1026, sodium nitroprusside – Sigma S0501) dissolved in control solution (i.e. saline with 1% dimethyl sulphoxide (DMSO – Sigma 276855) added to improve skin wetting). Comparison was also made to a commercially available NO donor ointment (Care Pharmaceuticals, Sydney) containing 0.2% wt/wt of the NO donor glyceryl trinitrate (GTNO). The test agents were applied over the rat hind limb within 1 minute after dye or venom injection with agents reapplied at ∼15 min intervals. The limb was wrapped with a thin layer of tissue to ensure the leg remained exposed to the agent for all experiments and maintained at a temperature of 35±1°C. Two types of experiments were performed. The first group of experiments tested the agent's effectiveness in slowing lymph transport by measuring the arrival time of a marker dye (India ink) in surgically exposed groin lymphatics consequent to injection of 50 µl of the dye into the corresponding hind foot of anaesthetized rats. The second group of experiments tested the agent's effectiveness in increasing the time to respiratory arrest of hind foot injection of Ptx venom.

Respiration frequency was measured visually or by recording chest movement through a strain gauge connected externally to the rat chest at the level of the diaphragm. Some experiments were made while simultaneously recording blood pressure and heart rate sampling data at 1 kHz (AD Instruments; Australia). Analysis of the rate of venom-induced decline of respiration frequency was generally made from the time of venom injection. In a minority of animals there was an initial increase in respiration rate and in these cases the respiration frequency was analyzed relative to its initial plateau.

Statistical significance confirmed by one-way ANOVA followed by Holm-Sidak's multiple comparison test. Data are presented as means ± s.e.m. with n referring to the number of animals.

## Results

### Pharmacological agents that slow the transit of lymph

**Figure 1:**
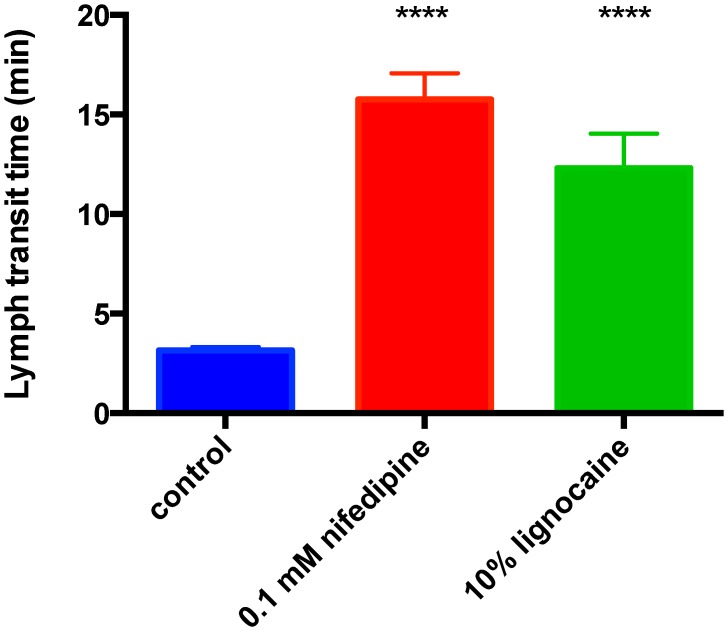
Effectiveness of the L-Ca channel blocker nifedipine and the local anesthetic lignocaine in inhibiting lymph flow in the rat hind limb. Foot to groin lymph transit times were measured using the marker dye India ink injected into a hind foot of each anaesthetized rat under control conditions or when the corresponding hind limb was covered with the topical pharmacological agent. The pharmacological agents all significantly increased transit times compared to control. Asterisks indicate significant differences with respect to control obtained by one-way ANOVA followed by Holm-Sidak's multiple comparison test (**** P<0.0001; n = 11, 10 & 6 for control, nifedipine & lignocaine respectively).

The effectiveness of two topical pharmacological agents, the Ca^2+^ channel antagonist nifedipine and the local anesthetic lignocaine at slowing the transit of lymph was tested. Nifedipine (0.1 mM) or lignocaine (10%) applied topically within 1 minute after rat hind foot dye injection, significantly (P<0.0001) slowed the transit of lymph, increasing the hind limb transit time of lymph by 500 and 390% respectively (Fig. 1).

### Effects of venom dose

**Figure 2:**
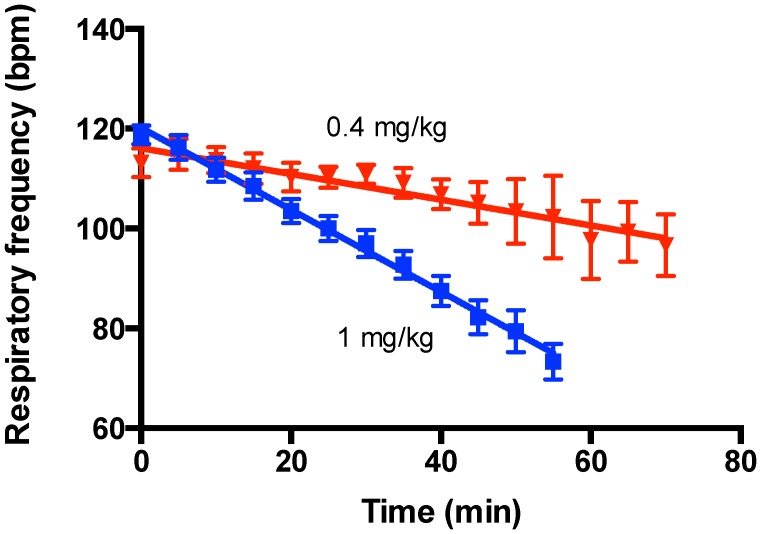
Effect of Ptx venom dose on rat respiration rate. Venom injected into a hind foot of each anaesthetized rat caused an approximately linear decline in respiration frequency before sudden arrest with the slope of the decline shallower at the lower venom dose (n = 16 & 7 for 1 mg/kg & 0.4 mg/kg respectively).

**Figure 3:**
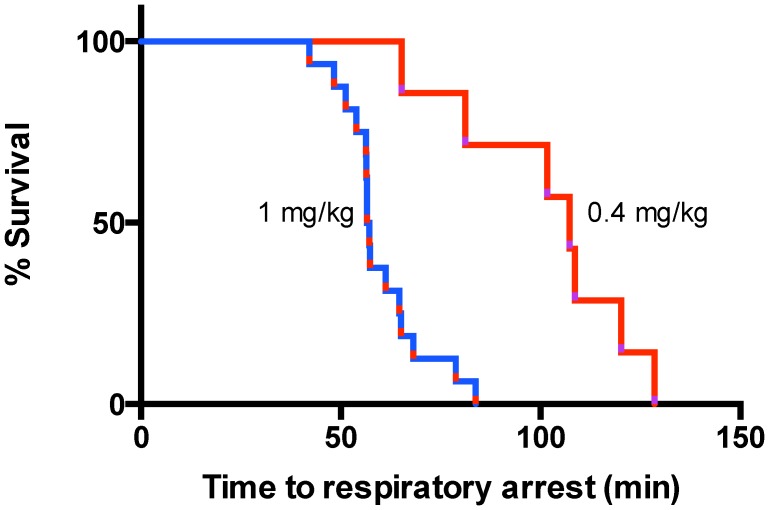
Effect of Ptx venom dose on the time to respiratory arrest consequent to hind foot injection in urethane anaesthetized rats.

**Figure 4:**
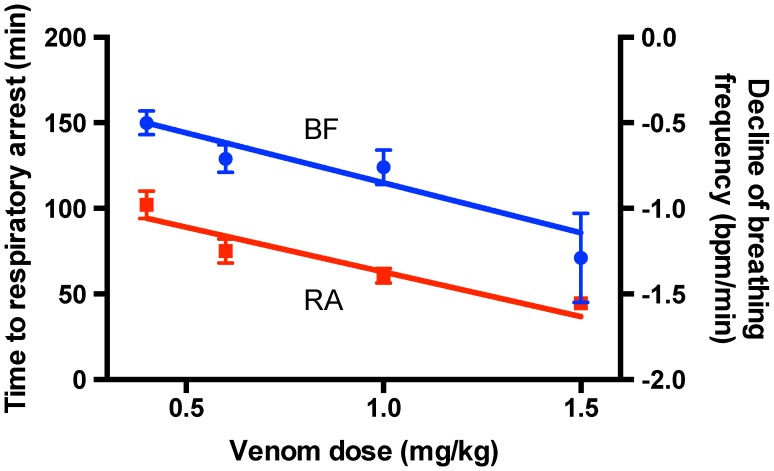
Summary plots of the mean times to respiratory arrest and mean venom-induced decline of respiration frequency for a range of Ptx venom doses. Both measures exhibited an approximately linear relationship with venom dose for the range shown (n = 6, 6, 11 & 3 for 0.4, 0.6, 1.0 & 1.5 mg/kg respectively).

Simulation of snakebite by injection of Ptx venom into the feet of deeply anaesthetized rats caused a linear reduction in respiration frequency. The rate of decline was reasonably constant over duration of the experiment and was dependent on venom dose with lower venom doses resulting in a slower/shallower decline in respiration frequency. Linear regression of the mean respiratory frequency plotted as a function of time for venom doses of 1.0 and 0.4 mg/kg provided respective slopes and intercepts of −0.82±0.05 & −0.26±0.05 bpm/min and 120±1 & 116±2 min (Fig. 2). Consistent with this, reducing the venom dose increased the time to respiratory arrest (Fig. 3). Both parameters showed an approximately linear dependence with venom dose over the range 1.5 to 0.4 mg/kg with respective slopes of −53±7 min/(mg/kg) and −0.6±0.1 bpm/min/(mg/kg) and intercepts of 115±7 min and −0.3±0.1 bpm/min for the time to respiratory arrest and the rate of venom-induced decline of the respiration frequency (Fig. 4). Rats survived at lower venom doses (range studied 0.1–0.2 mg/kg, n = 8) for the 2–4 h periods measured. At such doses, rats exhibited a small decrease in respiration rate which then recovered.

### Effectiveness of the pharmacological agents in slowing the onset of envenoming

**Figure 5:**
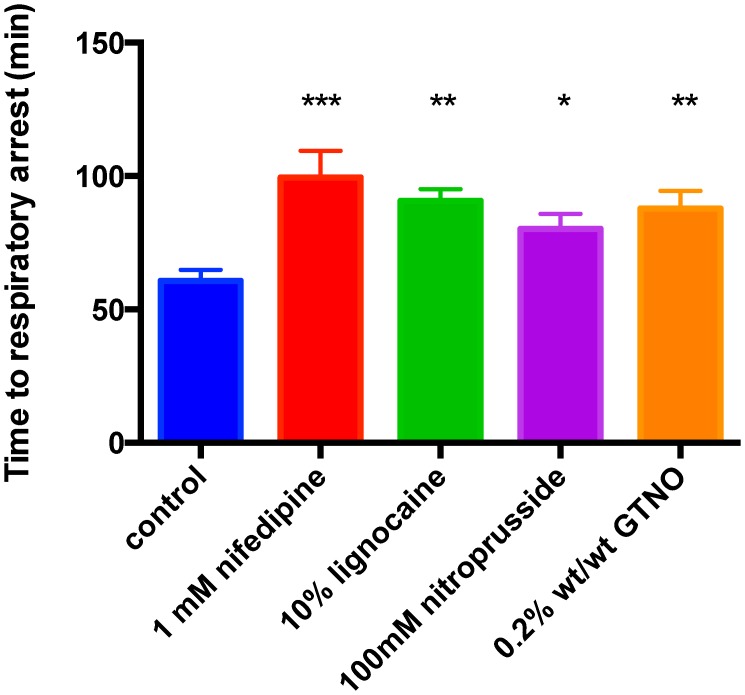
Summary bar plots of the mean times to respiratory arrest under control and test conditions for several pharmacological agents applied topically at the concentrations noted following injection of brown snake venom at the equivalent of 1/kg. All agents caused significant increase in time to respiratory arrest compared to control. Asterisks indicate significant differences with respect to control obtained by Mann Whitney test (* p<0.05, ** P<0.01, *** p<0.001; n = 11, 8, 5, 5 & 7 for control, nifedipine, lignocaine, 100 mM sodium nitroprusside and 0.2% wt/wt glyceryl trinitrate ointment respectively).

The effects of topical application of nifedipine, lignocaine or control solution (1% DMSO in saline) to hind limbs were examined by measuring the time to respiratory arrest and the rate of decline of the respiration frequency in anaesthetized rats consequent to foot injection of Ptx venom. Topical application of nifedipine (1 mM) and lignocaine (10%) significantly increased the time to respiratory arrest by 61% and 50% (Fig. 5). The NO donor sodium nitroprusside when applied at 10 mM or 100 mM caused a similar increase in time to respiratory arrest (75±7 min; n = 7 and 80±6 min, n = 5). In preliminary studies we observed that lower doses of nifedipine and lignocaine were similarly effective as those shown at higher doses in figure 5. Specifically, the time to respiratory arrest for nifedipine at 0.1 mM as studied on 2 animals was 87 and 95 min compared to 99±10 min (n = 8) for 1 mM nifedipine. The time to respiratory arrest for lignocaine at 5% as also studied on 2 animals was 90 and 80 min compared to 91±4 (n = 5) for 10% lignocaine. Studies comparing blood pressure or heart rate during application of Ptx venom (equivalent concentration - 1 mg/kg) under control (n = 4) and one of the experimental conditions (topical 1 mM nifedipine; n = 8) indicate there was no significant difference in heart rate and systolic or diastolic blood pressures between test and control data or during the recording period before respiratory arrest.”

The proportional effect of lymphatic inhibition by the topical agent was not dependent on the venom concentration and hence time of venom action. For example, while the time period for respiratory arrest was longer being 75±7 min (n = 6) compared to 61±4 min (n = 11) for rats injected with a lower Ptx venom concentration (near 0.65 mg/kg compared to 1 mg/kg respectively), the degree of slowing afforded by topical hind limb application of GTNO ointment occurred at a proportionally longer time of 109±9 min (n = 6) compared to 88±7 min (n = 7), with corresponding ratios (i.e. +GTNO/−GTNO) which were very similar (1.44 vs. 1.45).

## Discussion

The pharmacological agents tested here all inhibited the lymphatic system, as shown by a 300 to 500% increase in lymph transit times and an approximately 50% increase in the time to respiratory arrest with simulated Ptx envenomation. These data build on our previous finding that topical application of the NO donor GTNO (0.2% wt/wt glyceryl trinitrate ointment) increased the foot to groin transit time of lymph and the time to respiratory arrest consequent to simulated snakebite by similar amounts [13].

The finding that these agents had the same effect despite targeting different mechanisms provides insight into lymphatic function and the role of the lymphatics in venom absorption. The release of NO from GTNO or nitroprusside acts through the stimulation of the soluble guanylate cyclase, formation of cyclic GMP and activation of protein kinase G, the latter causing reduction in intracellular Ca^2+^ concentration and a decrease in the sensitivity of the contractile system to Ca^2+^ [14]. The net effect is both smooth muscle relaxation and an inability to pace the muscle [15], [16], which disables the lymphatic pump. Nifedipine, inhibits lymphatic pumping by blocking L-type Ca^2+^ channels, the voltage dependent channels that underlie generation of action potentials, entry of Ca^2+^ and consequent lymphatic smooth muscle contraction [17]. Therefore, nifedipine most likely produces the same outcome as the NO donors, which is to directly inhibit the lymphatic pump. In contrast, the local anesthetic lignocaine will block nerve (i.e. Na^+^) -mediated action potentials [18]. This suggests that transmitter release, presumably dominantly from sympathetic nerves is a key factor in maintaining spontaneous activity of the lymphatics [19]. Neurotransmitters released from sympathetic nerves include noradrenaline (i.e. norepinephrine) and the co-transmitters neuropeptide Y and ATP [20], [21]. Therefore agents that inhibit all or at least the dominant sympathetic transmitter should also be useful as a topical first aid. Our preliminary experiments measuring lymph transit times using the α-adrenoceptor blocker phentolamine support this view (data not shown).

The finding that the different lymphatic inhibitors slowed but did not block venom entry could be due to several factors. First, it is to be noted that lymph drainage occurs through both superficial lymphatics, which lie just under the skin and the deep lymphatic vessels, which drain the limb musculature and other deep tissues. Thus while venom absorption by the lymphatics will generally first occur through the superficial lymphatics following snakebite from short fanged snakes (i.e. most elapids), anastomoses also allow entry of lymph into the deep lymphatics [22]. Therefore, inhibition of lymph flow in the superficial lymphatics will not entirely block active lymph flow, which may still be mediated by the deep lymphatics, these being less influenced by the topical inhibitors. Second, there may also be passive lymph flow as will arise if there is a net positive interstitium-lymphatic pressure gradient causing lymph to flow centrally. Such flow will be aided by factors such as arterial pulsations, fluctuations of central venous pressure and skeletal muscle contractions [23], though there was no evidence of the latter in these deeply anaesthetized rats. Third, there may be low-level permeability of the vasculature to venom toxins, allowing entry of the toxins directly into the circulation.

The effects of venom dose were also examined with time to respiratory arrest found to approximately linearly decrease with increase in venom dose over the lethal dose range of 0.4 to 1.5 mg/kg studied. The fact that there was a relatively sharp cut off in the dose response such that venom doses of 0.2 mg/kg or less were not lethal whereas doses of 0.4 mg/kg or higher were always lethal highlights the importance of first aid procedures that slow and preferably limit venom entry into the circulation. It is also interesting that there wasn't a dose dependency in the effects of the pharmacological agents tested, although this was only tested in a small number of animals for lignocaine and nifedipine. This is likely to be because these doses for all three agents were already maximally inhibiting the lymphatics.

The study approach of using anaesthetized rats ensured that there was no muscle movement and hence no confounding of the data through activation of the extrinsic lymphatic pump. It also obviated ethical issues related to envenoming conscious animals. Some anesthetics have an effect on the level of sympathetic nervous activity and hence lymph flow rate. It was for this reason we chose to use urethane, as it increases sympathetic nervous activity [24], [25] and hence better represents circumstances where a snakebite victim remains still but because of fear has elevated sympathetic nerve activity. Importantly, in our previous publication on this subject, we found that GTNO ointment proportionally slowed lymphatic flow in conscious humans to the same extent as in urethane-anesthetized rats (see Fig. 1; [13]). Another observation we now make is that lymphatic inhibition had a proportional effect independent of the time course of venom action (see Results). Therefore, even if intrinsic lymphatic flow rate is modified by the anesthetic, the topical agents should still have a proportional effect and hence their effectiveness can be reasonably assessed.

### Concluding remarks

The key outcome of these studies is that a range of pharmacological agents known to directly or indirectly inhibit lymphatic pumping may be of use as topical treatments in first aid for bites from snakes whose venoms are not highly cytotoxic and where death by central action is the primary concern. It is suggested that they be considered as adjunct first aid to mechanical methods such as PBI (a snakebite first aid recommended by the Australian National Health and Medical Research Council), which while highly effective for limb bites are often incorrectly applied [10]. The topical agents might also be useful for bites to the torso and hence might be used as adjuncts to the local pressure pad with compression approach, which as indicated from animal studies is effective even for such bites [7]. Our previously reported findings for NO releasing ointment [13] indicate that a reasonable approach would be to apply an ointment formulation of the inhibitor just above the bite site. PBI or a local pressure pad with compression would then be applied. Finally, while all the compounds used in this study are used on humans for other purposes, the use of a local anesthetic is probably the most compelling, as it has the added advantage of reducing pain and hence may be readily adopted. However if used then the formulation should be rapidly acting, which was not the case for the commercially available formulations we tested (unpublished), but is the case for a commercially available NO releasing ointment [13].
